# How Students’ Perceptions of Physical Education Teachers’ Teaching Style Relate to Prosocial and Antisocial Behavior Among Chinese High School Students: The Role of Perceived Teacher-Supportive Instructional Climate

**DOI:** 10.3390/bs16101744

**Published:** 2026-09-24

**Authors:** Shuangrui Liu, Seungwoo Choi, Ansu Lee

**Affiliations:** 1 Department of Physical Education, Kyungpook National University, Daegu 41566, Republic of Korea; lsr980324@knu.ac.kr (S.L.); choisw75@knu.ac.kr (S.C.); 2 Institute of Student Physical Health Promotion, Jingchu University of Technology, Jingmen 448000, China

**Keywords:** physical education, teaching style, perceived teacher-supportive instructional climate, prosocial behavior, antisocial behavior, self-determination theory

## Abstract

Physical education provides an important context for adolescents’ social development because students regularly interact with teachers and peers through cooperation, competition, and shared learning experiences. Although supportive teaching has been associated with a range of positive educational outcomes, limited research has examined how multidimensional teaching style is related to students’ prosocial and antisocial behavior through students’ perceptions of a teacher-supportive instructional climate in Chinese secondary-school PE. This cross-sectional study examined the relationships among students’ perceptions of PE teachers’ teaching style, perceived teacher-supportive instructional climate (PTSIC), and prosocial and antisocial behavior in 710 Chinese high school students. Teaching style was conceptualized as a multidimensional construct comprising autonomy support, structure, and involvement. PTSIC was assessed using the 15-item Sport Climate Questionnaire (SCQ), and prosocial and antisocial behavior were assessed using the Prosocial and Antisocial Behavior in Sport Scale. Confirmatory factor analysis and structural equation modeling were conducted, and indirect associations were examined using bias-corrected bootstrapping with 2000 resamples. Teaching style was positively associated with PTSIC (β = 0.486, *p* < 0.001). PTSIC was positively associated with prosocial behavior (β = 0.422, *p* < 0.001) and negatively associated with antisocial behavior (β = −0.175, *p* < 0.001). Teaching style was negatively associated with antisocial behavior (β = −0.261, *p* < 0.001), whereas the specified model showed no corresponding direct association with prosocial behavior (β = 0.073, *p* = 0.157). Positive and negative indirect associations through PTSIC were observed for prosocial behavior (β = 0.205, 95% CI [0.153, 0.264]) and antisocial behavior (β = −0.085, 95% CI [−0.141, −0.035]), respectively. These findings suggest a pattern in which students who perceived more supportive multidimensional teaching also tended to perceive a more teacher-supportive instructional climate and report more favorable social behavior. Given the cross-sectional design and the study’s methodological limitations, we should interpret these findings as preliminary structural associations within the specified model rather than evidence of temporal ordering or causal mediation.

## 1. Introduction

Adolescence is an important developmental period for learning how to cooperate with others, manage interpersonal conflict, respond to social expectations, and regulate behavior across diverse social situations. Schools provide a central context for this development because students repeatedly interact with teachers and peers within structured environments governed by shared rules, expectations, and patterns of participation. Among school subjects, physical education (PE) offers a particularly visible and socially interactive setting in which students cooperate, compete, communicate, solve problems, evaluate performance, and respond to success and failure (Bailey et al., 2009; Casey & Goodyear, 2015; Gano-Overway, 2013; United Nations Educational, Scientific and Cultural Organization [UNESCO], 2015). These recurring interactions provide opportunities for adolescents to demonstrate helping, encouragement, cooperation, fairness, exclusion, hostility, and aggression. Accordingly, PE can be understood not only as a context for physical learning but also as an environment in which students’ social behavior is expressed and associated with their ongoing instructional and interpersonal experiences.

Prosocial and antisocial behaviors represent two conceptually distinct dimensions of adolescents’ social functioning. Prosocial behavior refers to voluntary actions intended to support or benefit others, such as helping, encouraging, cooperating, and showing respect. Such behavior is associated with positive peer relationships, social adjustment, and constructive participation in school life (El Mallah, 2020; Penner et al., 2005). Antisocial behavior includes actions that may harm, exclude, intimidate, or disadvantage others, including aggression, cheating, verbal hostility, and unfair conduct (Hodge & Gucciardi, 2015; Kavussanu & Boardley, 2009). Although prosocial and antisocial behaviors are generally negatively related, they should not necessarily be viewed as opposite endpoints of a single continuum. Previous research suggests that they may be associated with partly different motivational, interpersonal, and contextual processes (Hodge & Gucciardi, 2015; Hodge & Lonsdale, 2011; Kavussanu & Boardley, 2009). Accordingly, prosocial and antisocial behavior warrant separate consideration when examining their associations with instructional and social conditions.

Students’ social behavior is influenced not only by individual characteristics but also by the social environments in which interaction takes place (Busching & Krahé, 2020; Eccles & Roeser, 2011; Wentzel et al., 2010). In PE lessons, teachers play an important role in organizing these environments. They determine how activities are structured, communicate expectations, form groups, provide feedback, regulate competition, and respond to conflict or inappropriate conduct (Kulinna & Cothran, 2003; Mosston & Ashworth, 2008). Through these recurring practices, teachers may contribute to students’ perceptions of whether the learning environment is respectful, fair, supportive, pressuring, or controlling. Understanding how teacher behavior is associated with students’ social behavior therefore requires paying attention not only to specific instructional practices but also to the broader psychological environment that students perceive and experience during lessons.

Teaching style refers to relatively consistent patterns of teacher behavior involving instructional organization, communication, feedback, classroom management, and interpersonal interaction (Chatoupis, 2018; Mosston & Ashworth, 2008). In the present study, teaching style is conceptualized as a multidimensional construct comprising autonomy support, structure, and involvement (Skinner & Belmont, 1993). Autonomy support refers to practices that acknowledge students’ perspectives, provide meaningful choices, and explain the rationale for learning activities. Structure involves clear expectations, guidance, organization, and constructive feedback. Involvement refers to the extent to which teachers express interest, care, and interpersonal support. Together, these dimensions represent recurring instructional and interpersonal behaviors that may be associated with how students perceive and participate in PE lessons.

Self-Determination Theory (SDT) provides a relevant framework for understanding these processes. SDT proposes that social environments support adaptive functioning when they facilitate the basic psychological needs for autonomy, competence, and relatedness (Deci & Ryan, 2000; Ryan & Deci, 2020). Teachers who acknowledge students’ perspectives, provide clear guidance, offer constructive feedback, and foster supportive relationships may contribute to learning experiences perceived as more need-supportive and psychologically safe (Reeve, 2009; Reeve & Jang, 2006; Vansteenkiste et al., 2020). In PE, such teaching practices have been associated with motivation, engagement, enjoyment, well-being, and physical activity participation (Cheon et al., 2012; Cheon et al., 2016; Sparks et al., 2017). Supportive teaching may also be relevant to social behavior because students’ interactions with classmates occur within learning environments that are structured and regulated by the teacher.

However, the associations between students’ perceptions of teaching style and their social behavior may also be considered in relation to how students evaluate the broader supportive quality of the instructional environment created by the teacher. Repeated experiences of teacher behavior may contribute to students’ overall perceptions of whether PE lessons are understanding, respectful, supportive, and conducive to meaningful participation (Gairns et al., 2015). Such perceptions concern the supportive quality of the teacher-created instructional environment rather than classroom climate in its broadest sense, which may also encompass relational, organizational, and developmental characteristics extending beyond teacher support (Moos, 1973; Thapa et al., 2013).

Perceptions of the teacher-created supportive instructional environment are conceptually related to students’ perceptions of teaching style and may share some content, particularly with respect to teacher support. Nevertheless, the two can be distinguished in terms of the level and focus of students’ appraisal. Teaching style refers to students’ perceptions of recurring teacher behaviors across the dimensions of autonomy support, structure, and involvement (Skinner & Belmont, 1993). By contrast, perceptions of the supportive instructional environment reflect a more global appraisal of the extent to which the environment created by the teacher is understanding, supportive, and conducive to meaningful and self-directed participation (Deci & Ryan, 2000).

This distinction is therefore one of level and focus of appraisal rather than complete separation of content. Students may perceive, for example, that a teacher provides meaningful choices, communicates expectations clearly, offers constructive feedback, and demonstrates interpersonal concern. Across repeated lessons, these perceptions may be associated with a more general sense that the instructional environment created by the teacher is supportive (Warburton, 2017). Accordingly, the present study examines teaching style and PTSIC as theoretically related but non-interchangeable perceptions, while recognizing that some conceptual overlap between the constructs is possible.

Students’ perceptions of a supportive instructional environment may be particularly relevant to students’ prosocial and antisocial behavior in PE because lessons often involve public performance, physical contact, competition, peer comparison, and group dependence. Students who perceive the instructional environment created by their teacher as supportive and respectful may also report more constructive interactions with classmates. Conversely, perceptions of a less supportive instructional environment may be associated with greater hostility, unfair conduct, or other antisocial responses. Research in educational settings has linked positive school or classroom climates with greater school engagement and well-being (Lombardi et al., 2019) and with lower levels of bullying or peer victimization (Freeman et al., 2009; Thornberg et al., 2024). Although these studies generally adopt broader conceptualizations of classroom climate than the supportive instructional environment considered here, they provide relevant evidence that students’ perceptions of their educational environment are associated with social functioning.

Research on PE also provides evidence that autonomy-supportive and need-supportive teaching is associated with more adaptive classroom environments and social outcomes. Teacher-focused interventions have shown that autonomy-supportive teaching may improve students’ perceptions of classroom climate, strengthen prosocial peer processes, enhance prosocial behavior, and reduce antisocial behavior (Cheon et al., 2018; Cheon et al., 2019; Cheon et al., 2022; Cheon et al., 2023). These intervention findings provide theoretical support for examining whether students’ perceptions of teacher behavior, their broader perceptions of the supportive instructional environment, and their social behavior are systematically related. The present cross-sectional study does not test the temporal sequence suggested by these intervention findings; rather, it examines whether the theoretically specified pattern of associations is observed among Chinese high school students in PE.

First, previous studies have frequently focused on autonomy support as the primary dimension of teaching behavior. Recent PE research has also highlighted the importance of considering structure alongside autonomy-related dimensions when examining students’ motivation and engagement outcomes (Coterón et al., 2024). Nevertheless, less attention has been given to a multidimensional conception of teaching style that simultaneously incorporates autonomy support, structure, and involvement. Examining these dimensions together may provide a broader representation of the instructional and interpersonal behaviors that students experience in PE. Second, prosocial and antisocial behaviors represent distinct dimensions of social functioning, making it important to examine their respective patterns of association with multidimensional teaching style and perceptions of the supportive instructional environment within the same structural model. Third, much of the existing evidence has been generated through intervention studies or in educational contexts outside mainland Chinese secondary schools. Consequently, it remains unclear whether students’ perceptions of multidimensional teaching style and the supportive instructional environment are systematically associated with both forms of social behavior in Chinese high school PE.

China has increasingly emphasized moral education, holistic development, and students’ physical and psychological well-being through school PE (CPC Central Committee & State Council, 2020; General Office of the CPC Central Committee & General Office of the State Council, 2020). Recent discussions of PE reform have likewise highlighted efforts to move beyond narrowly performance-oriented approaches toward more comprehensive educational experiences that support students’ physical, psychological, and social development (Bashir et al., 2026). Nevertheless, teacher-directed instructional practices and strong teacher authority continue to characterize some Chinese educational contexts (Fang & Gopinathan, 2009). Previous research has also suggested that the relationships among autonomy support, psychological need satisfaction, and adaptive outcomes may vary across cultural PE contexts, including between Chinese and Western students (Taylor & Lonsdale, 2010). Within this educational context, students’ perceptions of autonomy support, structure, and teacher involvement may be particularly relevant to how they evaluate the social and psychological quality of PE lessons. Although research on motivational climate, need-supportive teaching, and life-skills development in Chinese PE has expanded in recent years, limited evidence is available on how multidimensional teaching style and students’ perceptions of the supportive instructional environment are jointly associated with students’ prosocial and antisocial behavior (Ji et al., 2025; Primo et al., 2023; Zheng et al., 2023).

The present study therefore examined the structural relationships among students’ perceptions of PE teachers’ teaching style, their perceptions of the supportive instructional environment created by the teacher, and students’ prosocial and antisocial behavior in Henan Province, China. Teaching style was conceptualized as a multidimensional construct consisting of autonomy support, structure, and involvement. For clarity, students’ broader perceptions of the supportive quality of the instructional environment created by their PE teacher are referred to in the present study as a perceived teacher-supportive instructional climate (PTSIC). The study examined theoretically specified direct and indirect associations among teaching style, PTSIC, and students’ social behavior. Because the data were cross-sectional, these indirect associations were interpreted as statistically specified structural associations rather than evidence of temporal ordering or causal mediation. This study extends SDT-informed research in three respects. First, it examines multidimensional teaching style rather than autonomy support alone. Second, it simultaneously examines the respective associations of prosocial and antisocial behavior with teaching style and PTSIC. Third, it investigates these relationships in the context of Chinese high school PE, where evidence remains limited.

Based on SDT and the preceding theoretical and empirical evidence, the following hypotheses were proposed:

**H1.**  *Teaching style is positively associated with PTSIC.*

**H2.**  *Teaching style is positively associated with prosocial behavior.*

**H3.**  *Teaching style is negatively associated with antisocial behavior.*

**H4.**  *PTSIC is positively associated with prosocial behavior.*

**H5.**  *PTSIC is negatively associated with antisocial behavior.*

**H6.**  *A statistically significant positive indirect association between teaching style and prosocial behavior is expected through PTSIC.*

**H7.**  *A statistically significant negative indirect association between teaching style and antisocial behavior is expected through PTSIC.*

The hypothesized model is presented in Figure 1.

## 2. Materials and Methods

### 2.1. Study Design

A cross-sectional survey was conducted to examine the structural relationships among students’ perceptions of PE teachers’ teaching style, PTSIC, and prosocial and antisocial behavior. Guided by SDT, the hypothesized relationships were examined using structural equation modeling (SEM), with a focus on the direct associations between teaching style and students’ social behavior and the statistical indirect associations through PTSIC. Because all study variables were measured at a single time point, the indirect paths were interpreted as theoretically specified statistical associations rather than evidence of temporal ordering or causal mediation (Maxwell & Cole, 2007). The hypothesized model was evaluated following the two-step approach proposed by Anderson and Gerbing (1988), in which the adequacy of the measurement models was first assessed through confirmatory factor analysis (CFA), followed by testing of the structural model (Kline, 2023).

### 2.2. Participants and Procedure

Participants were recruited from five public high schools in Henan Province, China, using convenience sampling. Approximately 150 students participated from each school. Recruitment was not based on the selection of intact PE classes. Rather, with permission from the school principals, PE teachers informed eligible students about the opportunity to participate, and participation was voluntary. Three PE teachers at each school assisted with recruitment, with a total of fifteen PE teachers across the five schools. The participating schools typically had approximately 20–28 classes, with approximately 50–60 students per class, and PE teachers generally taught multiple classes (approximately 10 classes each). Students were recruited across grade levels and classes rather than from a predetermined set of intact classes. Therefore, the exact number of classes represented in the analytic sample and class-level membership were not recorded as separate identifiers.

The target sample size was determined before data collection using an SEM sample-size calculation. An initial minimum sample of approximately 200 participants was estimated assuming a medium effect size, α = 0.05, and statistical power of 0.80. To conservatively account for potential clustering in the school-based sampling context, a design effect of approximately 3.37 was applied based on an assumed intraclass correlation of 0.03 and an average cluster size of approximately 80 students, resulting in a target of approximately 674 valid responses. Allowing for approximately 10% nonresponse or unusable questionnaires, at least 750 questionnaires were planned for distribution. The final analytic sample of 710 students exceeded the prespecified target of 674 valid responses.

Following approval from the Institutional Review Board of Kyungpook National University (approval no. KNU 2025-0748), permission to conduct the study was obtained from the participating schools. Before completing the survey, students and their legal guardians provided written informed consent. They were also advised that taking part in the study was voluntary, that their responses would be handled anonymously and confidentially, and that they were free to discontinue participation at any point without penalty.

Data were collected using anonymous paper-and-pencil questionnaires. Students completed the questionnaires individually and returned them in sealed envelopes. PE teachers assisted only with the collection of the sealed envelopes and did not open the envelopes or have access to individual students’ responses. The sealed questionnaires were subsequently transferred to the research team for data processing.

A total of 768 questionnaires were collected. In accordance with the prespecified data-screening criterion, questionnaires with an item-completion rate below 95% were excluded from the analysis. Questionnaires showing clearly invalid response patterns, such as straight-lining across items, were also excluded. Of the 58 excluded questionnaires, 7 were excluded because of insufficient item completion and 51 because of straight-lining response patterns. This resulted in a final analytic sample of 710 students (valid response rate = 92.4%). The final sample comprised 366 boys (51.5%) and 344 girls (48.5%). Regarding grade level, 212 students (29.9%) were in Grade 10, 241 (33.9%) were in Grade 11, and 257 (36.2%) were in Grade 12.

### 2.3. Measures

Because the instruments were originally developed in English, they were translated and culturally adapted for use with Chinese high school students before data collection. A multistage procedure involving translation, back-translation, expert review, adjudication, and pretesting was used to promote linguistic clarity and conceptual equivalence. First, the original English items were translated into Chinese by a bilingual expert with doctoral-level qualifications and relevant expertise in PE and educational psychology. An independent bilingual expert in the same fields subsequently produced an English back-translation of the Chinese questionnaire. This version was reviewed against the source instrument to check for differences in intended meaning. The two experts then jointly reviewed any inconsistencies, unclear wording, or contextually unsuitable expressions and agreed on revisions to the Chinese version.

The preliminary Chinese-language questionnaire was subsequently pretested with 36 Chinese high school students to evaluate item clarity, comprehensibility, and contextual appropriateness. Some expressions that students found difficult to understand were revised based on their feedback to improve clarity and comprehensibility. Adaptations were intended to preserve the conceptual meaning of the original items while ensuring their relevance to Chinese high school PE contexts.

#### 2.3.1. Teaching Style

Students’ perceptions of PE teachers’ teaching style were assessed using the Teacher as Social Context Questionnaire (TASC-Q; Skinner & Belmont, 1993). In the present study, teaching style refers to the extent to which students perceived their PE teacher as providing autonomy support, structure, and involvement during lessons. The TASC-Q consists of twenty-four items across three subscales, namely autonomy support, structure, and involvement, with eight items for each subscale. Autonomy support reflects the degree to which teachers acknowledge students’ perspectives and provide meaningful opportunities for choice and self-directed participation. Structure refers to the extent to which teachers provide clear expectations, guidance, feedback, and organization for learning activities. Involvement reflects students’ perceptions that teachers show interest, care, and interpersonal support. Accordingly, the TASC-Q was used to assess students’ perceptions of recurring and relatively specific teacher behaviors rather than their global evaluation of the classroom environment.

All items were rated on a five-point Likert scale ranging from 1 (strongly disagree) to 5 (strongly agree). Prior to analysis, nine negatively worded items were reverse-coded (Items 3, 4, 6, 7, 11, 12, 18, 20, and 24), so that higher scores consistently indicated a more supportive teaching style. The overall scale demonstrated excellent internal consistency in the present sample (Cronbach’s α = 0.957).

#### 2.3.2. Perceived Teacher-Supportive Instructional Climate

Students’ perceptions of the teacher-supportive instructional climate in PE were assessed using the 15-item Sport Climate Questionnaire (SCQ; Deci & Ryan, 2000). In the present study, the construct assessed by the SCQ is referred to as perceived teacher-supportive instructional climate (PTSIC) to reflect its teacher-focused scope and to distinguish it from broader conceptualizations of classroom climate. PTSIC represents students’ global appraisal of the extent to which the instructional environment created by their PE teacher is understanding, supportive, and conducive to meaningful and self-directed participation. Accordingly, whereas the TASC-Q assesses students’ perceptions of recurring teacher behaviors across autonomy support, structure, and involvement, the SCQ was used to assess students’ broader evaluation of the supportive quality of the teacher-created instructional environment.

The SCQ consists of 15 items assessing students’ perceptions of the extent to which their teacher understands their perspectives, provides interpersonal support, and facilitates meaningful and self-directed participation. Responses were recorded on a seven-point Likert scale ranging from 1 (strongly disagree) to 7 (strongly agree). Item 13 was reverse-coded before analysis, and higher scores indicated a more teacher-supportive instructional climate. A composite score was calculated by averaging responses across the 15 items. The scale demonstrated excellent internal consistency in the present sample (Cronbach’s α = 0.949).

#### 2.3.3. Prosocial and Antisocial Behavior

Students’ social behavior during PE lessons was assessed using an adapted version of the Prosocial and Antisocial Behavior in Sport Scale (PABSS; Kavussanu & Boardley, 2009). Prosocial behavior was defined as positive interpersonal behavior intended to support or benefit others, including helping, encouraging, cooperating with, and showing respect toward peers. Antisocial behavior referred to negative interpersonal actions, including verbal hostility, exclusion, unfair conduct, and behaviors that could adversely affect classmates’ participation experiences. The PABSS consists of 20 items representing four subscales: prosocial behavior toward teammates (4 items), prosocial behavior toward opponents (3 items), antisocial behavior toward teammates (5 items), and antisocial behavior toward opponents (8 items).

Because the original scale was developed for organized sport, minor linguistic adaptations were made to enhance its applicability to the PE context. The original teammate- and opponent-referenced wording was retained in the Chinese version. No additional standardized instructions were provided to redefine “teammates” or “opponents” across different types of PE activities. The adapted wording was reviewed as part of the translation and cultural adaptation procedure to preserve the intended meaning of the original items. Following previous PE-based applications of the PABSS (Cheon et al., 2018), the items were rated using a seven-point Likert response scale ranging from 1 (strongly disagree) to 7 (strongly agree). Higher scores indicated stronger endorsement of the corresponding prosocial or antisocial behavior. In the present sample, the Cronbach α coefficients for the four subscales ranged from 0.931 to 0.956, indicating excellent internal consistency.

### 2.4. Data Analysis

Data were analyzed using SPSS version 29.0 (IBM Corp., Armonk, NY, USA) and AMOS version 29.0 (IBM Corp., Armonk, NY, USA). Descriptive statistics, including means, standard deviations, skewness, and kurtosis, were calculated to evaluate the distributional characteristics of the measured variables. Internal consistency was assessed using Cronbach’s α coefficients, and Pearson correlation coefficients were calculated to examine bivariate associations among the study variables.

The analysis proceeded at two related but distinct measurement levels. First, item-level CFAs were conducted to evaluate the factorial validity of the TASC-Q and PABSS. Second, subscale mean scores were used as indicators in the structural model to provide a more parsimonious representation of the constructs while retaining their theoretically defined multidimensional structure.

For teaching style, an item-level second-order CFA was conducted using the 24 TASC-Q items. Autonomy support, structure, and involvement were specified as first-order factors, and teaching style was specified as a second-order latent factor. This higher-order specification reflected the theoretical conceptualization of autonomy support, structure, and involvement as distinct but related dimensions of an overarching supportive teaching style.

For students’ social behavior, an item-level CFA was conducted using the 20 PABSS items. Prosocial behavior toward teammates, prosocial behavior toward opponents, antisocial behavior toward teammates, and antisocial behavior toward opponents were specified as four correlated first-order factors, consistent with the four theoretically defined dimensions of the PABSS.

PTSIC was treated as an observed composite variable by averaging the 15 SCQ items. The composite score was used as the operational measure of students’ overall perceived teacher-supportive instructional climate in the structural model. Because PTSIC was modeled as an observed composite, measurement error in this construct was not estimated separately in the structural model.

In the structural model, teaching style was represented as a latent construct indicated by the mean scores of autonomy support, structure, and involvement. Prosocial behavior and antisocial behavior were represented as latent constructs indicated by their corresponding PABSS subscale mean scores, whereas PTSIC was entered as an observed composite variable. Because prosocial and antisocial behavior were each represented by two subscale indicators, the loading of the teammate indicator was fixed to 1.00 for each latent construct to set its measurement scale, while the loading of the opponent indicator and the indicator residual variances were freely estimated. Prosocial and antisocial behavior were specified as distinct endogenous outcomes, and their residual disturbances were not allowed to covary because no residual association between these outcomes was specified a priori in the hypothesized model. Thus, the item-level CFAs were used to establish the factorial validity of the TASC-Q and PABSS, whereas the subsequent SEM used theoretically defined subscale scores as indicators of the latent constructs and the SCQ composite score as the observed measure of PTSIC.

Convergent validity was examined based on standardized factor loadings, composite reliability (CR), and average variance extracted (AVE), where applicable. Because PTSIC was modeled as an observed composite rather than a latent construct, conventional latent-variable tests of discriminant validity were not applicable to PTSIC. Correlations between PTSIC and the teaching-style dimensions were therefore examined to characterize the degree of empirical association between the measures, but they were not interpreted as establishing discriminant validity. These associations were interpreted in light of the a priori conceptual distinction between students’ perceptions of recurring teacher behaviors and their more global appraisal of the supportive quality of the teacher-created instructional environment, while recognizing the possibility of conceptual and method-related overlap between the measures.

SEM with maximum likelihood estimation was subsequently performed to test the hypothesized relationships among teaching style, PTSIC, and students’ prosocial and antisocial behavior. The indirect associations of teaching style with prosocial and antisocial behavior through PTSIC were examined using bias-corrected bootstrapping with 2000 resamples and 95% confidence intervals. Evidence of an indirect association was inferred from whether the resulting bootstrap interval excluded zero. Given the cross-sectional design, statistically significant indirect paths were interpreted as statistical indirect associations rather than evidence of causal mediation.

Model fit was assessed using the chi-square-to-degrees-of-freedom ratio (χ^2^/df), comparative fit index (CFI), Tucker–Lewis index (TLI), root mean square error of approximation (RMSEA), and standardized root mean square residual (SRMR). The 90% CI was also reported for RMSEA. CFI and TLI values ≥ 0.90 and RMSEA and SRMR values < 0.08 were used as benchmarks for acceptable fit (Hu & Bentler, 1999; Kline, 2023). GFI, AGFI, and NFI were additionally reported for the item-level measurement models. Statistical tests used an alpha level of 0.05, and structural associations were reported as standardized coefficients.

## 3. Results

### 3.1. Descriptive Statistics and Correlations

Descriptive statistics and bivariate correlations among the study variables are reported in Table 1. The absolute values of skewness ranged from 0.077 to 0.629, whereas kurtosis values ranged from −1.206 to −0.490. These values indicated no substantial departures from univariate normality and supported the use of maximum likelihood estimation in the subsequent analyses (Kline, 2023).

The three dimensions of teaching style were moderately to strongly intercorrelated (*r* = 0.569–0.575, *p* < 0.01). PTSIC was positively correlated with autonomy support (*r* = 0.341, *p* < 0.01), structure (*r* = 0.392, *p* < 0.01), and involvement (*r* = 0.369, *p* < 0.01). These moderate correlations indicated that PTSIC was related to, but not highly correlated with, the three teaching-style dimensions; however, these correlations were not interpreted as establishing discriminant validity. PTSIC was also positively correlated with prosocial behavior toward teammates (*r* = 0.346, *p* < 0.01) and opponents (*r* = 0.371, *p* < 0.01) and negatively correlated with antisocial behavior toward teammates (*r* = −0.198, *p* < 0.01) and opponents (*r* = −0.251, *p* < 0.01). Similarly, autonomy support, structure, and involvement showed positive correlations with both prosocial behavior dimensions and negative correlations with both antisocial behavior dimensions. Overall, the bivariate correlations were generally consistent with the hypothesized directions of association.

### 3.2. Measurement Models

Prior to testing the structural model, item-level CFAs were conducted to evaluate the factorial validity of the study measures. Because PTSIC was operationalized as an observed composite variable in the structural model, the item-level measurement-model analyses reported here focused on teaching style and students’ social behavior.

A second-order CFA was conducted for teaching style using the 24 TASC-Q items. Autonomy support, structure, and involvement were specified as first-order factors, and teaching style was specified as a second-order latent factor. The model demonstrated good fit (χ^2^(249) = 457.300, χ^2^/df = 1.837, GFI = 0.949, AGFI = 0.939, NFI = 0.967, TLI = 0.983, CFI = 0.985, RMSEA = 0.034, 90% CI [0.029, 0.039], SRMR = 0.024). The standardized second-order factor loadings were acceptable, ranging from 0.751 to 0.760. The CR was 0.800, and the AVE was 0.572. These findings supported the representation of autonomy support, structure, and involvement as dimensions of a higher-order teaching style construct.

A correlated four-factor CFA was conducted for the 20-item PABSS, with prosocial behavior toward teammates, prosocial behavior toward opponents, antisocial behavior toward teammates, and antisocial behavior toward opponents specified as correlated first-order factors. The model showed a good fit to the data (χ^2^(164) = 230.575, χ^2^/df = 1.406, GFI = 0.968, AGFI = 0.959, NFI = 0.983, TLI = 0.994, CFI = 0.995, RMSEA = 0.024, 90% CI [0.016, 0.031], SRMR = 0.018). Standardized factor loadings ranged from 0.802 to 0.960 across the four factors, supporting the factorial validity of the four-factor measurement structure.

As summarized in Table 2, the measurement-model results supported the reliability and convergent validity of the teaching-style construct and the four first-order social-behavior dimensions. Overall, these results provided adequate support for their use in the subsequent structural analysis.

### 3.3. Structural Model

The structural model demonstrated acceptable fit to the data (χ^2^(16) = 67.296, χ^2^/df = 4.206, CFI = 0.969, TLI = 0.945, RMSEA = 0.067, 90% CI [0.051, 0.084], SRMR = 0.064). Teaching style was positively associated with PTSIC (β = 0.486, *p* < 0.001). The direct association between teaching style and prosocial behavior was small (β = 0.073, *p* = 0.157), whereas teaching style was negatively associated with antisocial behavior (β = −0.261, *p* < 0.001). PTSIC was positively associated with prosocial behavior (β = 0.422, *p* < 0.001) and negatively associated with antisocial behavior (β = −0.175, *p* < 0.001). The standardized path coefficients are presented in Figure 2 and Table 3.

### 3.4. Indirect-Association Analysis

The indirect associations through PTSIC were examined using bias-corrected bootstrapping with 2000 resamples. Teaching style showed a positive indirect association with prosocial behavior through PTSIC (β = 0.205, 95% CI [0.153, 0.264], *p* < 0.001), with the CI not including zero; this pattern was consistent with H6. The corresponding direct association (β = 0.073, 95% CI [−0.037, 0.181]) had a CI including zero, whereas the total association was positive, with its CI not including zero (β = 0.278, 95% CI [0.182, 0.370]).

Teaching style also showed a negative indirect association with antisocial behavior through PTSIC (β = −0.085, 95% CI [−0.141, −0.035], *p* < 0.001), with the CI not including zero; this pattern was consistent with H7. The corresponding direct association (β = −0.261, 95% CI [−0.377, −0.135]) and total association (β = −0.346, 95% CI [−0.444, −0.242]) were also negative, with their CIs not including zero. Given the cross-sectional design, these findings represent concurrent patterns of direct and indirect association within the specified structural model rather than evidence of a temporal mediation process. The standardized total, direct, and indirect associations are summarized in Table 4.

## 4. Discussion

The present study examined the structural relationships among students’ perceptions of PE teachers’ teaching style, PTSIC, and high school students’ prosocial and antisocial behavior within Chinese secondary school PE. Grounded in SDT, the study examined theoretically specified direct and indirect associations among perceived teaching style, PTSIC, and students’ social behavior. The findings showed that perceived teaching style was positively associated with PTSIC and that PTSIC was positively associated with prosocial behavior and negatively associated with antisocial behavior. Perceived teaching style was also directly and negatively associated with antisocial behavior, whereas no corresponding direct association with prosocial behavior was evident within the specified model. In addition, positive and negative indirect associations through PTSIC were observed for prosocial and antisocial behavior, respectively. These findings suggest a pattern in which students’ perceptions of multidimensional teaching style, their broader appraisal of the teacher-supportive instructional environment, and their reported social behavior were systematically related within the specified structural model. Given the cross-sectional design, however, these findings should be interpreted as preliminary concurrent structural associations rather than evidence that perceived teaching style temporally precedes PTSIC or that PTSIC causally mediates subsequent changes in students’ social behavior.

One observed pattern was that students’ perceptions of teaching style were positively associated with PTSIC. This finding is consistent with SDT, which emphasizes the role of teachers’ instructional and interpersonal behaviors in shaping students’ experiences within the learning environment (Ryan & Deci, 2020; Vansteenkiste et al., 2020). In PE, teachers organize learning tasks, communicate expectations, provide feedback, regulate peer interactions, and respond to students’ emotional and behavioral needs. Students may therefore perceive these recurring teaching practices as part of their broader experience of the instructional environment. The positive association observed between teaching style and PTSIC is consistent with this theoretical expectation. Importantly, however, the finding should be interpreted with recognition that both constructs represent students’ perceptions of teacher-related experiences and may share some content. Teaching style captures students’ perceptions of recurring teacher behaviors across autonomy support, structure, and involvement, whereas PTSIC reflects a more global appraisal of the supportive quality of the instructional environment created by the teacher. Thus, the distinction between the two constructs concerns primarily the level and focus of students’ appraisal rather than a complete separation of content.

This distinction does not imply that teaching style and PTSIC are empirically independent or completely non-overlapping constructs. In the present study, PTSIC showed moderate correlations with autonomy support, structure, and involvement (r = 0.341–0.392), indicating that the measures were related but not highly correlated. Nevertheless, because both constructs were assessed through students’ perceptions and both include teacher-supportive elements, some conceptual and method-related overlap remains possible. Accordingly, the present findings should not be interpreted as demonstrating complete empirical separation between teaching style and PTSIC. Rather, they are consistent with the proposed conceptual distinction between relatively specific perceptions of recurring teaching behaviors and a more global appraisal of the supportive quality of the teacher-created instructional environment, while recognizing that these perceptions are theoretically and empirically related.

Perceived teaching style showed different patterns of association with prosocial and antisocial behavior. For prosocial behavior, no direct association with teaching style was evident within the specified model, whereas PTSIC was positively associated with prosocial behavior. In addition, teaching style showed a positive indirect association with prosocial behavior through PTSIC. This pattern suggests that, within the specified model, the association between perceived teaching style and prosocial behavior was evident in the specified indirect path through students’ broader appraisal of the teacher-supportive instructional environment, whereas the corresponding direct association was not evident. Prosocial behavior in PE often occurs in situations requiring cooperation, communication, shared responsibility, and responsiveness to classmates (Casey & Goodyear, 2015; Gano-Overway, 2013). Students who perceive the instructional environment created by their PE teacher as supportive may therefore also tend to report greater helping, encouragement, cooperation, and respect toward peers. Importantly, however, the observed indirect association does not establish that perceived teaching style temporally shaped PTSIC or that PTSIC subsequently produced prosocial behavior.

In contrast, perceived teaching style was directly and negatively associated with antisocial behavior even after PTSIC was included in the model, and a negative indirect association through PTSIC was also observed. Thus, unlike the pattern for prosocial behavior, the association between teaching style and antisocial behavior was observed in both direct and indirect paths within the specified structural model. PE lessons frequently involve competition, physical contact, public performance, and peer comparison, which may create situations in which conflict or inappropriate conduct can occur (Gano-Overway, 2013; Hodge & Gucciardi, 2015). In such situations, students who perceive greater autonomy support, structure, and involvement from their PE teacher may also report lower levels of antisocial behavior, while their broader appraisal of the teacher-supportive instructional environment is additionally associated with lower antisocial behavior (Montero-Carretero & Cervelló, 2019). Because teaching style was modeled as a multidimensional higher-order construct, the direct association with antisocial behavior should not be attributed to any single teaching-style dimension. Rather, it represents the association of the broader configuration of perceived autonomy support, structure, and involvement with antisocial behavior.

Taken together, the findings indicate different patterns of statistical association for prosocial and antisocial behavior. Prosocial behavior showed an indirect association with teaching style through PTSIC without a corresponding direct association being evident, whereas antisocial behavior showed both direct and indirect associations with teaching style. However, these differences should not be interpreted as evidence of distinct causal mechanisms. The study did not formally test whether the corresponding structural coefficients differed significantly across the two behavioral outcomes, and the cross-sectional design does not establish temporal ordering among teaching style, PTSIC, and students’ social behavior. Longitudinal or experimental research is therefore needed to determine whether these observed patterns reflect distinct developmental or instructional processes.

One contribution of this study lies in examining PTSIC as a theoretically specified linking construct within the structural associations between perceived teaching style and students’ social behavior. Previous SDT-based research in PE has often emphasized motivation, psychological need satisfaction, engagement, enjoyment, and physical activity participation (Cheon et al., 2012; Cheon et al., 2016; Sparks et al., 2017). The present study builds on this line of research by applying an SDT-informed framework to prosocial and antisocial behavior. Importantly, the model distinguishes between students’ perceptions of recurring teaching behaviors and their more global appraisal of the teacher-created supportive instructional environment, while recognizing that these perceptions are conceptually related and may partially overlap.

The study also contributes by conceptualizing teaching style as a multidimensional construct comprising autonomy support, structure, and involvement rather than focusing on autonomy support alone. This approach recognizes that supportive PE teaching may involve providing meaningful choice, clarifying expectations, offering constructive feedback, organizing participation, and demonstrating interpersonal care. Accordingly, the observed associations reflect a broader configuration of perceived supportive teaching rather than the contribution of any single teaching-style dimension.

A further contribution is the simultaneous examination of prosocial and antisocial behavior. The findings showed different patterns of association for the two outcomes: perceived teaching style was not directly associated with prosocial behavior but was directly and negatively associated with antisocial behavior, whereas PTSIC was associated with both outcomes. This pattern is consistent with previous research suggesting that prosocial and antisocial behaviors may be related to partly distinct motivational and contextual processes (Hodge & Gucciardi, 2015; Hodge & Lonsdale, 2011; Kavussanu & Boardley, 2009). However, this difference should not be overstated. The study did not formally test whether the corresponding structural coefficients differed significantly across the two outcomes. Thus, the findings indicate different patterns of statistical association but do not establish that the underlying processes are statistically or causally distinct.

These findings are also relevant to the context of Chinese secondary school PE. Recent policy discussions have emphasized more holistic approaches to students’ physical, psychological, moral, and social development through school PE (Bashir et al., 2026). The present findings indicate that students’ perceptions of supportive teaching and the teacher-supportive instructional environment are associated with their reported social behavior within this context. However, because the study did not include cross-cultural or regional comparisons, the findings should not be interpreted as demonstrating that these associations are unique to Chinese educational culture. Cultural explanations therefore remain theoretical possibilities rather than empirically tested conclusions.

### 4.1. Theoretical and Practical Implications

From a theoretical perspective, the findings contribute to SDT-informed research in PE by highlighting the relevance of students’ perceptions of supportive teaching not only to motivational and learning-related experiences but also to their reported social behavior. The findings further suggest the value of distinguishing between students’ perceptions of recurring teaching behaviors and their broader appraisal of the teacher-supportive instructional environment. At the same time, because teaching style and PTSIC are conceptually related and were both assessed through students’ self-reports, this distinction should be understood in terms of the level and focus of appraisal rather than as evidence of complete empirical separation. Future research using longitudinal, observational, or experimental designs could examine whether these constructs show distinct predictive relationships with students’ social behavior over time.

From a practical perspective, the findings suggest that PE teachers may benefit from considering how their recurring instructional and interpersonal practices are experienced by students as part of the broader supportive quality of the instructional environment. Practices such as providing meaningful choices, explaining the rationale for activities, offering constructive feedback, acknowledging students’ perspectives, and demonstrating consistent interpersonal involvement are theoretically consistent with supportive teaching and may contribute to meaningful participation. However, because the present study was cross-sectional, these findings should inform pedagogical reflection rather than be interpreted as evidence that adopting particular teaching practices will necessarily produce changes in students’ social behavior. Intervention research nevertheless provides complementary evidence that autonomy-supportive and interpersonally supportive teaching can contribute to more adaptive PE experiences (Cheon et al., 2012; Cheon et al., 2016; Sparks et al., 2017).

Drawing on the broader PE literature, teachers may consider learning opportunities that encourage students to communicate, assist one another, rotate roles, solve problems collaboratively, and reflect on respectful participation. Cooperative learning, peer-assisted tasks, group reflection, inclusive team activities, and structured opportunities to support less-skilled classmates provide pedagogical contexts in which prosocial interaction can occur (Casey & Goodyear, 2015; Gano-Overway, 2013). These strategies were not examined directly in the present study and should therefore be regarded as literature-informed pedagogical recommendations rather than empirically demonstrated effects of the current findings.

The negative associations between perceived teaching style and antisocial behavior also highlight the potential relevance of clear and supportive instructional structure. Within an SDT-informed approach, teachers can communicate behavioral expectations, explain the rationale for rules, respond consistently to inappropriate conduct, and organize competitive activities in ways that emphasize predictability, guidance, and respect rather than coercion or humiliation (Reeve, 2009; Vansteenkiste et al., 2020). Such practices may provide conditions that are less conducive to aggression, exclusion, disrespect, and unfair conduct, although this possibility was not directly tested in the present study. Moreover, because teaching style was modeled as a multidimensional higher-order construct, its association with antisocial behavior cannot be attributed specifically to structure or to any other individual teaching-style dimension.

Finally, the findings have implications for PE teacher education and professional development. Teacher education in PE has traditionally emphasized core instructional competencies, including lesson planning, instructional implementation, observation, evaluation, and supervised practicum experiences (Derri et al., 2015). In light of the present findings and the broader literature, teacher education may also benefit from more explicit attention to communication, feedback practices, peer-group organization, conflict management, responses to exclusion or hostility, and the design of learning tasks that provide opportunities for constructive peer interaction. Such training may also encourage teachers to consider how students experience recurring instructional and interpersonal practices as part of the broader supportive quality of the teacher-created instructional environment.

### 4.2. Limitations and Future Directions

Several limitations should be acknowledged. First, the cross-sectional design prevents causal inference regarding the relationships among teaching style, PTSIC, and students’ social behavior. Although the proposed model was grounded in SDT and previous empirical research, the temporal sequence among the variables cannot be confirmed, and reciprocal relationships remain possible (Maxwell & Cole, 2007). In addition, unmeasured individual, relational, and institutional factors—including peer relationships, personality traits, moral disengagement, competitive orientation, family background, achievement pressure, school culture, and broader policy expectations—may partly account for the observed associations. Future longitudinal studies incorporating relevant covariates, as well as intervention studies, are needed to examine temporal relationships more rigorously.

Second, all variables were assessed using student self-report measures. Although students’ perceptions are theoretically important, the observed relationships may partly reflect shared method variance, response tendencies, social desirability, or generalized evaluations of the PE teacher and class. This issue is particularly relevant to teaching style and PTSIC because both constructs were based on student perceptions and included teacher-supportive content. Their correlations do not establish discriminant validity, and some conceptual and method-related overlap cannot be ruled out. Future research should examine their empirical distinctiveness using latent-variable and multimethod designs incorporating student reports, teacher reports, and classroom observations.

Third, PTSIC was treated as an observed composite variable based on the SCQ. This approach supported model parsimony but did not estimate measurement error separately. Consequently, measurement error in PTSIC may have affected the magnitude of the estimated structural associations involving this construct, and these path estimates should therefore be interpreted with appropriate caution. In addition, the SCQ primarily captures students’ perceptions of teacher-provided autonomy support and interpersonal support rather than the broader multidimensional characteristics often encompassed by the concept of classroom climate. For this reason, the construct was operationalized in the present study as PTSIC, and the findings should be interpreted specifically in relation to this teacher-focused supportive instructional appraisal. Future research could model PTSIC as a latent construct and examine its factorial structure and measurement properties more directly. Studies addressing classroom climate more broadly should employ measures that also capture peer relationships, psychological safety, fairness, participation norms, cohesion, and classroom organization.

Fourth, the participants were recruited from public high schools in Henan Province using convenience sampling, and the hierarchical structure of students nested within classes, teachers, and schools was not explicitly modeled. The findings may therefore have limited generalizability, and the assumption of independent observations may have been violated, potentially affecting the estimated standard errors, confidence intervals, and significance tests. Although a design effect was used for conservative sample-size planning, this adjustment did not correct for potential clustering in the SEM itself. Accordingly, the individual path estimates and associated inferential statistics should be interpreted cautiously. Future research should use more diverse samples and multilevel designs to distinguish individual-level perceptions from shared class- or teacher-level characteristics.

Fifth, autonomy support, structure, and involvement were modeled as dimensions of a higher-order teaching-style construct. Although this specification was consistent with the study’s focus on multidimensional teaching style, the three dimensions are theoretically distinguishable and may show different associations with students’ social behavior. Modeling them as a higher-order construct may therefore have obscured dimension-specific associations with prosocial and antisocial behavior. Future research should examine these dimensions separately to determine whether they show distinct patterns of association with students’ social behavior.

Sixth, several measurement limitations concern the assessment of prosocial and antisocial behavior. The PABSS retains teammate- and opponent-referenced items from organized sport, and students participating in competitive, cooperative, or individual PE activities may have interpreted these terms differently. In addition, prosocial and antisocial behavior were each represented by only two subscale indicators in the structural model, limiting the evaluation of their measurement structure despite model identification through a fixed marker loading. Future research should examine PE-specific adaptations of the PABSS and consider alternative measurement models or broader assessments of social behavior.

Seventh, the study did not formally compare the magnitudes of the structural paths for prosocial and antisocial behavior. The observed differences therefore indicate different descriptive patterns but do not establish that the relevant associations were statistically different. Future studies could use parameter-constraint tests to compare these paths directly.

Taken together, these methodological limitations—including potential unmodeled clustering, conceptual and method-related overlap between teaching style and PTSIC, concurrent self-report measurement, the observed-composite treatment of PTSIC, and uncertainty surrounding the interpretation of teammate- and opponent-referenced PABSS items—limit the strength of inference that can be drawn from the individual structural paths. Accordingly, the present findings should be viewed as preliminary structural associations within the specified model and require replication using designs that more directly address these methodological constraints.

## 5. Conclusions

The present study examined associations among students’ perceptions of PE teachers’ teaching style, PTSIC, and prosocial and antisocial behavior in a sample of high school students from Henan Province, China. Perceived teaching style was positively associated with PTSIC, which was positively associated with prosocial behavior and negatively associated with antisocial behavior. Perceived teaching style was also directly and negatively associated with antisocial behavior, whereas no corresponding direct association with prosocial behavior was evident within the specified model. Positive and negative indirect associations through PTSIC were observed for prosocial and antisocial behavior, respectively.

These findings should be interpreted as preliminary individual-level, cross-sectional structural associations within the specified model and do not establish temporal ordering, causal mediation, or teacher- or classroom-level effects. Given the methodological limitations of the study, the individual path estimates and associated inferential statistics should be interpreted cautiously. Longitudinal and multilevel research is needed to clarify the temporal ordering of these associations and to distinguish individual-level perceptions from shared teacher- or classroom-level characteristics.

## Figures and Tables

**Figure 1:**
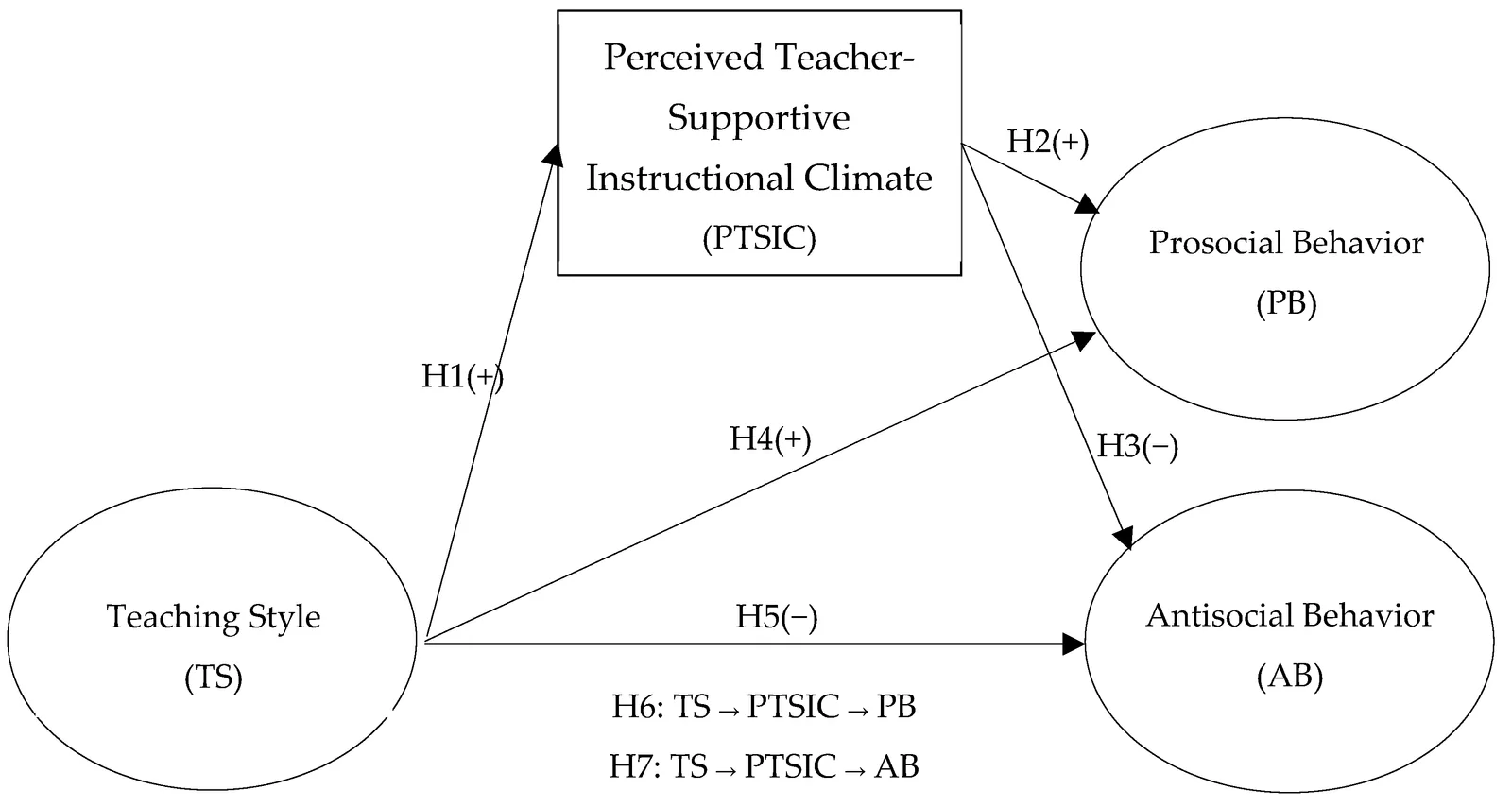
Hypothesized structural model.

**Figure 2:**
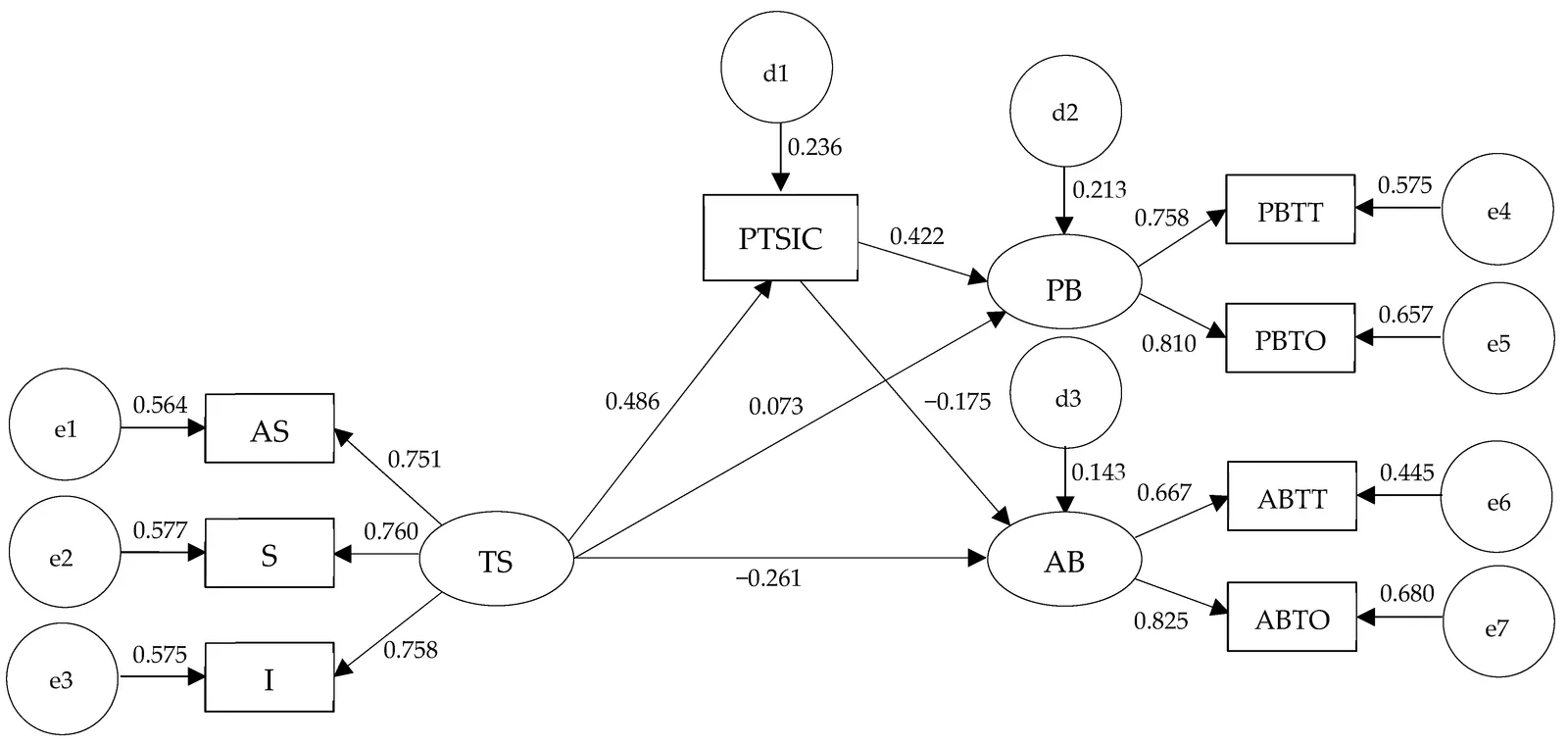
Structural model illustrating the standardized path coefficients among teaching style, perceived teacher-supportive instructional climate, and students’ prosocial and antisocial behavior. Note. TS = teaching style; PTSIC = perceived teacher-supportive instructional climate; PB = prosocial behavior; AB = antisocial behavior; AS = autonomy support; S = structure; I = involvement; PBTT = prosocial behavior toward teammates; PBTO = prosocial behavior toward opponents; ABTT = antisocial behavior toward teammates; ABTO = antisocial behavior toward opponents.

**Table 1:** Descriptive statistics and correlations among study variables (N = 710).

Variable	Mean	SD	Skewness	Kurtosis	1	2	3	4	5	6	7	8
1. AS	3.50	1.10	−0.279	−0.881	—							
2. S	3.49	1.12	−0.283	−0.918	0.569 **	—						
3. I	3.56	1.09	−0.403	−0.759	0.575 **	0.571 **	—					
4. PTSIC	4.28	1.52	−0.077	−0.861	0.341 **	0.392 **	0.369 **	—				
5. PBTT	4.59	1.80	−0.236	−10.128	0.175 **	0.197 **	0.158 **	0.346 **	—			
6. PBTO	4.48	1.88	−0.220	−10.206	0.184 **	0.182 **	0.161 **	0.371 **	0.614 **	—		
7. ABTT	3.08	1.63	−0.518	−0.642	−0.192 **	−0.164 **	−0.185 **	−0.198 **	0.111 **	0.067	—	
8. ABTO	3.00	1.60	−0.629	−0.490	−0.235 **	−0.204 **	−0.214 **	−0.251 **	0.130 **	0.082 *	0.550 **	—

Note. AS = autonomy support; S = structure; I = involvement; PTSIC = perceived teacher-supportive instructional climate; PBTT = prosocial behavior toward teammates; PBTO = prosocial behavior toward opponents; ABTT = antisocial behavior toward teammates; ABTO = antisocial behavior toward opponents. * *p* < 0.05, ** *p* < 0.01.

**Table 2:** Confirmatory factor analysis results and convergent validity.

Construct	Indicators	Standardized Loading	CR	AVE
Teaching Style	Autonomy Support	0.751	0.800	0.572
	Structure	0.760		
	Involvement	0.758		
Prosocial Behavior Toward Teammates	Item 1	0.906	0.939	0.794
	Item 2	0.886		
	Item 3	0.853		
	Item 4	0.917		
Prosocial Behavior Toward Opponents	Item 5	0.960	0.941	0.841
	Item 6	0.899		
	Item 7	0.892		
Antisocial Behavior Toward Teammates	Item 8	0.802	0.931	0.730
	Item 9	0.884		
	Item 10	0.839		
	Item 11	0.891		
	Item 12	0.858		
Antisocial Behavior Toward Opponents	Item 13	0.864	0.956	0.732
	Item 14	0.856		
	Item 15	0.846		
	Item 16	0.869		
	Item 17	0.860		
	Item 18	0.836		
	Item 19	0.851		
	Item 20	0.855		

**Table 3:** Structural path analysis results.

Path	B	β	SE	C.R.	*p*
TS → PTSIC (H1)	0.888	0.486	0.076	11.615	<0.001
TS → PB (H2)	0.121	0.073	0.085	1.414	0.157
TS → AB (H3)	−0.341	−0.261	0.080	−4.263	<0.001
PTSIC → PB (H4)	0.379	0.422	0.047	8.132	<0.001
PTSIC → AB (H5)	−0.125	−0.175	0.037	−3.357	<0.001

Note. TS = teaching style; PTSIC = perceived teacher-supportive instructional climate; PB = prosocial behavior; AB = antisocial behavior; β = standardized path coefficient; SE = standard error; C.R. = critical ratio.

**Table 4:** Standardized total, direct, and indirect associations of teaching style with students’ social behavior.

Outcome	Total β [95% CI]	Direct β [95% CI]	Indirect β [95% CI]
Prosocial Behavior	0.278 [0.182, 0.370]	0.073 [−0.037, 0.181]	0.205 [0.153, 0.264]
Antisocial Behavior	−0.346 [−0.444, −0.242]	−0.261 [−0.377, −0.135]	−0.085 [−0.141, −0.035]

Note. β = standardized effect; CI = confidence interval; PTSIC = perceived teacher-supportive instructional climate. Indirect associations were estimated through PTSIC. Confidence intervals were estimated using a bias-corrected bootstrap procedure with 2000 resamples.

## Data Availability

The data presented in this study are available on request from the corresponding authors due to privacy and ethical restrictions.

## Abbreviations

The following abbreviations are used in this manuscript:

PE	physical education
SDT	Self-Determination Theory
PTSIC	Perceived teacher-supportive instructional climate
SEM	structural equation modeling
CFA	confirmatory factor analysis
TASC-Q	Teacher as Social Context Questionnaire
SCQ	Sport Climate Questionnaire
PABSS	Prosocial and Antisocial Behavior in Sport Scale
CFI	comparative fit index
TLI	Tucker–Lewis index
RMSEA	root mean square error of approximation
SRMR	standardized root mean square residual
CR	composite reliability
AVE	average variance extracted

## References

[B1-behavsci-16-01744] Anderson J. C., Gerbing D. W. (1988). Structural equation modeling in practice: A review and recommended two-step approach. Psychological Bulletin.

[B2-behavsci-16-01744] Bailey R., Armour K., Kirk D., Jess M., Pickup I., Sandford R., BERA Physical Education and Sport Pedagogy Special Interest Group (2009). The educational benefits claimed for physical education and school sport: An academic review. Research Papers in Education.

[B3-behavsci-16-01744] Bashir M., Grecic D., Li Z., Tian J., Lu J., Liu Z., Li P. (2026). A new era for physical education in China: Teacher and pupil experiences and implications for the physical education profession. Frontiers in Education.

[B4-behavsci-16-01744] Busching R., Krahé B. (2020). With a little help from their peers: The impact of classmates on adolescents’ development of prosocial behavior. Journal of Youth and Adolescence.

[B5-behavsci-16-01744] Casey A., Goodyear V. A. (2015). Can cooperative learning achieve the four learning outcomes of physical education? A review of literature. Quest (National Association for Kinesiology in Higher Education).

[B6-behavsci-16-01744] Chatoupis C. C. (2018). Physical education teachers’ use of Mosston and Ashworth’s teaching styles: A literature review. The Physical Educator.

[B7-behavsci-16-01744] Cheon S. H., Reeve J., Lee Y., Ntoumanis N., Gillet N., Kim B. R., Song Y. (2019). Expanding autonomy psychological need states from two (satisfaction, frustration) to three (dissatisfaction): A classroom-based intervention study. Journal of Educational Psychology.

[B8-behavsci-16-01744] Cheon S. H., Reeve J., Marsh H. W. (2023). Autonomy-supportive teaching enhances prosocial and reduces antisocial behavior via classroom climate and psychological needs: A multilevel randomized control intervention. Journal of Sport & Exercise Psychology.

[B9-behavsci-16-01744] Cheon S. H., Reeve J., Marsh H. W., Song Y. (2022). Intervention-enabled autonomy-supportive teaching improves the PE classroom climate to reduce antisocial behavior. Psychology of Sport and Exercise.

[B10-behavsci-16-01744] Cheon S. H., Reeve J., Moon I. S. (2012). Experimentally based, longitudinally designed, teacher-focused intervention to help physical education teachers be more autonomy supportive toward their students. Journal of Sport & Exercise Psychology.

[B11-behavsci-16-01744] Cheon S. H., Reeve J., Ntoumanis N. (2018). A needs-supportive intervention to help PE teachers enhance students’ prosocial behavior and diminish antisocial behavior. Psychology of Sport and Exercise.

[B12-behavsci-16-01744] Cheon S. H., Reeve J., Song Y. (2016). A teacher-focused intervention to decrease PE students’ amotivation by increasing need satisfaction and decreasing need frustration. Journal of Sport & Exercise Psychology.

[B13-behavsci-16-01744] Coterón J., Fernández-Caballero J., Martín-Hoz L., Franco E. (2024). The interplay of structuring and controlling teaching styles in physical education and its impact on students’ motivation and engagement. Behavioral Sciences.

[B14-behavsci-16-01744] CPC Central Committee, State Council (2020). Overall plan for deepening educational evaluation reform in the new era *[Policy document]*.

[B15-behavsci-16-01744] Deci E. L., Ryan R. M. (2000). The “what” and “why” of goal pursuits: Human needs and the self-determination of behavior. Psychological Inquiry.

[B16-behavsci-16-01744] Derri V., Vasiliadou O., Kioumourtzoglou E. (2015). The effects of a short-term professional development program on physical education teachers’ behaviour and students’ engagement in learning. European Journal of Teacher Education.

[B17-behavsci-16-01744] Eccles J. S., Roeser R. W. (2011). Schools as developmental contexts during adolescence. Journal of Research on Adolescence.

[B18-behavsci-16-01744] El Mallah S. (2020). Conceptualization and measurement of adolescent prosocial behavior: Looking back and moving forward. Journal of Research on Adolescence.

[B19-behavsci-16-01744] Fang Y., Gopinathan S., Saha L. J., Dworkin A. G. (2009). Teachers and teaching in Eastern and Western schools: A critical review of cross-cultural comparative studies. International handbook of research on teachers and teaching.

[B20-behavsci-16-01744] Freeman J. G., Samdal O., Klinger D. A., Dur W., Griebler R., Currie D., Rasmussen M. (2009). The relationship of schools to emotional health and bullying. International Journal of Public Health.

[B21-behavsci-16-01744] Gairns F., Whipp P. R., Jackson B. (2015). Relational perceptions in high school physical education: Teacher- and peer-related predictors of female students’ motivation, behavioral engagement, and social anxiety. Frontiers in Psychology.

[B22-behavsci-16-01744] Gano-Overway L. A. (2013). Exploring the connections between caring and social behaviors in physical education. Research Quarterly for Exercise and Sport.

[B23-behavsci-16-01744] General Office of the CPC Central Committee, General Office of the State Council (2020). Opinions on comprehensively strengthening and improving school physical education in the new era.

[B24-behavsci-16-01744] Hodge K., Gucciardi D. F. (2015). Antisocial and prosocial behavior in sport: The role of motivational climate, basic psychological needs, and moral disengagement. Journal of Sport & Exercise Psychology.

[B25-behavsci-16-01744] Hodge K., Lonsdale C. (2011). Prosocial and antisocial behavior in sport: The role of coaching style, autonomous vs. controlled motivation, and moral disengagement. Journal of Sport & Exercise Psychology.

[B26-behavsci-16-01744] Hu L., Bentler P. M. (1999). Cutoff criteria for fit indexes in covariance structure analysis: Conventional criteria versus new alternatives. Structural Equation Modeling.

[B27-behavsci-16-01744] Ji X., Zheng S., Cheng L. (2025). Students’ perceptions of need-supportive teaching, basic psychological need satisfaction, and life skills development in Chinese physical education. Frontiers in Psychology.

[B28-behavsci-16-01744] Kavussanu M., Boardley I. D. (2009). The prosocial and antisocial behavior in sport scale. Journal of Sport & Exercise Psychology.

[B29-behavsci-16-01744] Kline R. B. (2023). Principles and practice of structural equation modeling.

[B30-behavsci-16-01744] Kulinna P. H., Cothran D. J. (2003). Physical education teachers’ self-reported use and perceptions of various teaching styles. Learning and Instruction.

[B31-behavsci-16-01744] Lombardi E., Traficante D., Bettoni R., Offredi I., Giorgetti M., Vernice M. (2019). The impact of school climate on well-being experience and school engagement: A study with high-school students. Frontiers in Psychology.

[B32-behavsci-16-01744] Maxwell S. E., Cole D. A. (2007). Bias in cross-sectional analyses of longitudinal mediation. Psychological Methods.

[B33-behavsci-16-01744] Montero-Carretero C., Cervelló E. (2019). Teaching styles in physical education: A new approach to predicting resilience and bullying. International Journal of Environmental Research and Public Health.

[B34-behavsci-16-01744] Moos R. H. (1973). Conceptualizations of human environments. American Psychologist.

[B35-behavsci-16-01744] Mosston M., Ashworth S. (2008). Teaching physical education.

[B36-behavsci-16-01744] Penner L. A., Dovidio J. F., Piliavin J. A., Schroeder D. A. (2005). Prosocial behavior: Multilevel perspectives. Annual Review of Psychology.

[B37-behavsci-16-01744] Primo L., González-Hernández J., Yang Y., López de Subijana C. (2023). Predicting social skills in disadvantaged Chinese high school students through physical education. Frontiers in Psychology.

[B38-behavsci-16-01744] Reeve J. (2009). Why teachers adopt a controlling motivating style toward students and how they can become more autonomy supportive. Educational Psychologist.

[B39-behavsci-16-01744] Reeve J., Jang H. (2006). What teachers say and do to support students’ autonomy during a learning activity. Journal of Educational Psychology.

[B40-behavsci-16-01744] Ryan R. M., Deci E. L. (2020). Intrinsic and extrinsic motivation from a self-determination theory perspective: Definitions, theory, practices, and future directions. Contemporary Educational Psychology.

[B41-behavsci-16-01744] Skinner E. A., Belmont M. J. (1993). Motivation in the classroom: Reciprocal effects of teacher behavior and student engagement across the school year. Journal of Educational Psychology.

[B42-behavsci-16-01744] Sparks C., Lonsdale C., Dimmock J., Jackson B. (2017). An intervention to improve teachers’ interpersonally involving instructional practices in high school physical education: Implications for student relatedness support and in-class experiences. Journal of Sport & Exercise Psychology.

[B43-behavsci-16-01744] Taylor I. M., Lonsdale C. (2010). Cultural differences in the relationships among autonomy support, psychological need satisfaction, subjective vitality, and effort in British and Chinese physical education. Journal of Sport & Exercise Psychology.

[B44-behavsci-16-01744] Thapa A., Cohen J., Guffey S., Higgins-D’Alessandro A. (2013). A review of school climate research. Review of Educational Research.

[B45-behavsci-16-01744] Thornberg R., Wänström L., Sjögren B., Bjereld Y., Edling S., Francia G., Gill P. (2024). A multilevel study of peer victimization and its associations with teacher support and well-functioning class climate. Social Psychology of Education.

[B46-behavsci-16-01744] United Nations Educational, Scientific and Cultural Organization (UNESCO) (2015). Quality physical education (QPE): Guidelines for policy-makers.

[B47-behavsci-16-01744] Vansteenkiste M., Ryan R. M., Soenens B. (2020). Basic psychological need theory: Advancements, critical themes, and future directions. Motivation and Emotion.

[B48-behavsci-16-01744] Warburton V. E. (2017). Peer and teacher influences on the motivational climate in physical education: A longitudinal perspective on achievement goal adoption. Contemporary Educational Psychology.

[B49-behavsci-16-01744] Wentzel K. R., Battle A., Russell S. L., Looney L. B. (2010). Social supports from teachers and peers as predictors of academic and social motivation. Contemporary Educational Psychology.

[B50-behavsci-16-01744] Zheng S., Ji X., Cheng L., Xu J., Cronin L. D. (2023). Perceptions of the motivational climate, basic psychological needs, and life skills development in Chinese physical education students. Frontiers in Psychology.

